# Broadband and Efficient Metamaterial Absorber Design Based on Gold–MgF2–Tungsten Hybrid Structure for Solar Thermal Application

**DOI:** 10.3390/mi14051066

**Published:** 2023-05-17

**Authors:** Ammar Armghan, Meshari Alsharari, Khaled Aliqab

**Affiliations:** Department of Electrical Engineering, College of Engineering, Jouf University, Sakaka 72388, Saudi Arabia

**Keywords:** broadband, finite element method, computational, solar thermal energy, absorber

## Abstract

We have presented a solar absorber design with gold–MgF_2_–tungsten materials. The solar absorber design is optimized with nonlinear optimization mathematical method to find and optimize geometrical parameters. The wideband absorber is made of a three-layer structure composed of tungsten, magnesium fluoride, and gold. This study analyzed the absorber’s performance using numerical methods over the sun wavelength range of 0.25 μm to 3 μm. The solar AM 1.5 absorption spectrum is a benchmark against which the proposed structure’s absorbing characteristics are evaluated and discussed. It is necessary to analyze the behavior of the absorber under a variety of various physical parameter conditions in order to determine the results and structural dimensions that are optimal. The nonlinear parametric optimization algorithm is applied to obtain the optimized solution. This structure can absorb more than 98% of light across the near-infrared and visible light spectrums. In addition, the structure has a high absorption efficiency for the far range of the infrared spectrum and the THz range. The absorber that has been presented is versatile enough to be used in a variety of solar applications, both narrowband and broadband. The design of the solar cell that has been presented will be of assistance in designing a solar cell that has high efficiency. The proposed optimized design with optimized parameters will help design solar thermal absorbers.

## 1. Introduction

The natural environment offers abundant resources that may be used to extract energy, which can be used for both conventional and renewable types of electricity. The use of energy derived from natural sources has the potential to make the utilization of renewable energy sources more socially acceptable. The primary objective of the research that is now being conducted is to identify some unique approaches that may be utilized to analyze naturally occurring resources. This endeavor aims to increase the utility of these resources and make the world a better place. In this research project, the sun’s rays are being analyzed in great detail as a possible renewable energy source. This solar collector was utilized by Sir John Herschel in order to bring his food to a boil. Edmond Becquerel, a French scientist, discovered the discovery of photovoltaic effects in 1898 when he was doing tests with an electrolytic cell, which is a device comprised of two electrodes that are placed in a conducting solution [1]. The cell produced a greater amount of power after being made to be exposed to light. Willoughby Smith soon after found that selenium has qualities that made it photoconductive. The researchers have already finished a portion of their study, in which they improved absorption qualities by working on varying forms of metamaterial structures. Structures such as double split-ring resonators (SRRs), U-shaped SRRs, chiral, thin wires, and a great number of others fall under this category [2,3,4,5,6].

Metallic square rings were used as the active component in producing a polarization-insensitive, four-band terahertz absorbable material. This material was created by using the rings. It has been found that the size of the metallic rings and the total number of rings contribute in some way to the resonance band [7]. A planar metamaterial perfect absorber may offer a wide-band response and wide-angle results [8,9,10,11,12]. This particular kind of absorber combines several different resonant components to manufacture it. It is feasible to make infrared absorbers by incorporating graphene-based structures. This process may be carried out in a factory. They have an absorption rate of up to 95% at large angles of incidence, and this rate is unaffected by the polarization of the light that is striking them [13]. Ultrathin chromium, when shaped into a refractory metal, can absorb 90 percent throughout the spectrum of wavelengths ranging from 0.4 to 1.4 μm [14]. By utilizing a less complicated design, a metamaterial can absorb more than 80% across a broad bandwidth in the THz domain [15,16]. Approximately 86.35% of the energy from the AM1.5 portion of the sun’s spectrum may be absorbed by nanorods with several layers and unique shapes [17]. It was conceivable to construct a unit cell with fourfold symmetry by applying the technique in specific circumstances when it was appropriate. It indicates polarization-insensitive behavior at wide angles and an absorption rate of more than 90% across the visible and infrared spectra [18]. Additionally, the spectrum shows that the material can absorb infrared light. Gold resonators provide high efficiency in the visible band for light that is incident at a different polarization like TE and TM polarization [19].

The optimization of the structure can be possible by using different parametric optimization algorithms. One of them is the nonlinear parametric optimization method which is used for the structures behaving nonlinearly concerning the wavelength or frequency. The nonlinear linear optimization is considered if: The functions are not behaving linearly which gives this optimization. It has function f(x), constraint $c_{i}\left(x\right)=1,2,\ldots \ldots \ldots n$ or $d_{j}\left(x\right)=1,2,\ldots \ldots \ldots n$ are components of x that are nonlinear [20]. The results of such structures are optimized for different parameters, such as design parameters. One such optimization is presented in solar absorber design presented by researchers recently [5,21]. The optimization methods improve the overall absorption of the different solar absorbers and also it can enhance the results of the other designs as well [22]. The nonlinear parametric optimization is applied when one parameter is dependent on the other and there is nonlinear behavior in their results.

The solar absorbers are applicable in solar energy harvesting applications [23] where the energy absorbed from the solar radiation is converted into heat which can be applied in solar water heaters [24]. The heat can also be used to generate electricity which can be used in day-to-day appliances or industrial needs [25].

Currently, the most critical design aim for solar cells is to increase their capacity to absorb light across a wide spectrum of wavelengths. The proposed research aims to improve wideband performance by utilizing a structure based on solar cells, and this project aims to improve broadband performance. Utilizing a ground plane made of tungsten helped reduce the absorber’s overall cost and adding a layer of magnesium fluoride (MgF_2_) helped increase the device’s capacity for absorbing radiation. Both factors helped to bring the total cost of the absorber down. To study the consequences that these modifications have on the response, various other features of the resonator are subjected to various kinds of alterations. Since gold is utilized in producing solar cells, the doors may react to frequencies in the near-visible and near-infrared regions of the electromagnetic spectrum. Solar energy has the potential to be transformed into several types of useful power. The findings of the solar cell’s broadband absorption are compared to its standard spectrum of absorption (AM 1.5) to determine how efficient the solar cell is. It is done so that the efficiency of the solar cell may be determined. It is done to understand how effective the solar cell will be. The current study highlights the impact of the solar absorber’s design on the physical features, and it does so by showing how the solar absorber was designed. It is also able to be utilized in the process of selecting the building of a solar absorber, which is another one of its capabilities.

## 2. Solar Absorber Design and Modeling

**Figure 1:**
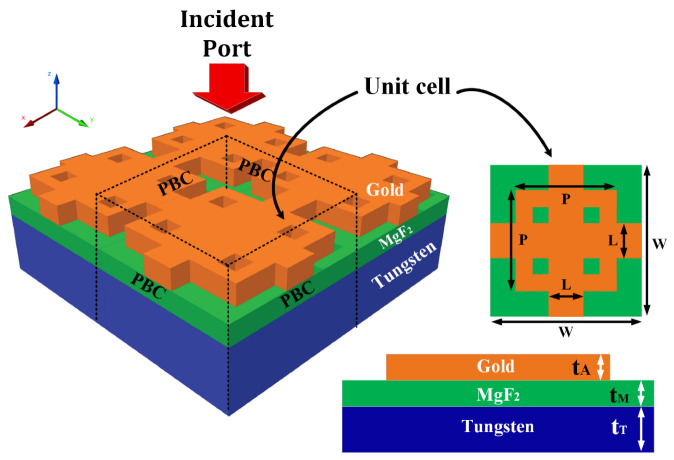
Schematic of the proposed solar absorber and its different views. The three-dimensional view of the solar absorber is on the left the top view is on the right top and the front view is on the right bottom. The hybrid structure of gold–MgF2–tungsten is presented in different views. t_A_ = 1000 nm, L = 300 nm, t_M_ = 1000 nm, t_T_ = 2500 nm, W = 2000 nm, P = 1000 nm.

The design of the metamaterial structure is given in Figure 1. The design is presented in the figure with different views. The right side of the figure shows the structure’s top and front views. This hybrid structure is formed using layers of tungsten, MgF_2_, and gold material. We have optimized all the parameter values by applying the optimization to all the parameters. The optimized values of all the parameters are listed here and in Table 1. The optimization of all the parameters is explained in this section. The top gold material is shaped according to the cross and squared structure. The overall structure’s dimension (W × W) is set as 2000 nm × 2000 nm. The height of the tungsten (t_T_), MgF_2_ (t_M_), and gold (t_A_) are set as 2500 nm, 1000 nm, and 1000 nm, respectively. The dimensions of the top layer are set as L = 250 nm and P = 1000 nm. The proposed construction for a solar absorber is given a numerical investigation in which periodic boundary conditions in the X and Y directions are considered. As shown in Figure 1, the incident wave travels down the *Z*-axis. The light reflection caused by the proposer absorber can be decreased with the assistance of the MgF_2_ layer. The gold resonator structure will act as a barrier for the light as it travels through it. Together, the gold and the MgF_2_ work to lower the reflectance and increase the absorption through the layers present in the design. The design parameters are listed in Table 1. All the design construction parameters are listed in the table.

The resonator structure developed on top of the substrate is crucial in achieving high absorption. To achieve a high absorption effect, the gold resonator plays a significant role. The equation that describes the effectiveness of gold-based absorption is given in Equations (1)–(5) [26], and it is derived from the following:

(1) $$A\left(\omega \right)=\frac{Q_{abs}\left(\omega \right)}{Q_{inc}\left(\omega \right)}$$

(2) Qabs=ωε02∫v0Im[ε(ω)]|E|2dV

(3) $$Q_{inc}=SF\left(\omega \right)$$

(4) $$Q_{abs}^{tot}=\int^{\;}A\left(\omega \right)F\left(\omega \right)d\omega$$

(5) A(ω)=1−T(ω)−R(ω)

The description of the structure is presented in the reference [26]. The absorption of the structure depends on the ratio of the absorbed power to the incident power. The absorption also depends on the amount transmitted and reflected through the structure. A broadband structure is quantitatively studied through the finite element method (FEM). PDEs are applied to find solutions for problems that are influenced by time and space. The vast majority of PDEs and geometries are challenging to solve using analytical methods. Instead, the approximation is derived by utilizing many distinct methods for dissecting equations. Equations contained within the numerical approach. When differential equations are approximated using structure equations then it is easy to solve them using the numerical approach. These design models can produce an estimate close to the correct response. One technology that is utilized for precise equivalency is FEM. The structural model for the predicted behavior is shown in Equation (6), below. The index of refraction of MgF_2_ is fixed at 1.375, whereas the index of refraction of tungsten is set at 1.24. The formation of the table is based on taking into account the material properties of the gold, as shown in [27]. The gold resonator in the shape of a cross is laid out in a pattern on top of the MgF_2_ to create an array, which boosts the structure’s capacity for absorption. According to [28,29], the formula for absorption is A=1−R−T, $T={\left|S_{21}\right|}^{2},$ and $R={\left|S_{11}\right|}^{2}$ are the formulas used to define the reflectance and transmittance coefficients, respectively.

(6) $$\nabla \times \mu_{r}^{-1}\left(\nabla \times E\right)-k_{0}^{2}\left(\epsilon_{r}-\frac{j\sigma }{\omega \epsilon_{0}}\right)E=0$$

## 3. Results and Discussion

In this section, the detailed investigation of the results of the hybrid structure presented in Figure 1 is carried out and the response is presented in different figures given in this section with their detailed discussion presented in this section.

**Figure 2:**
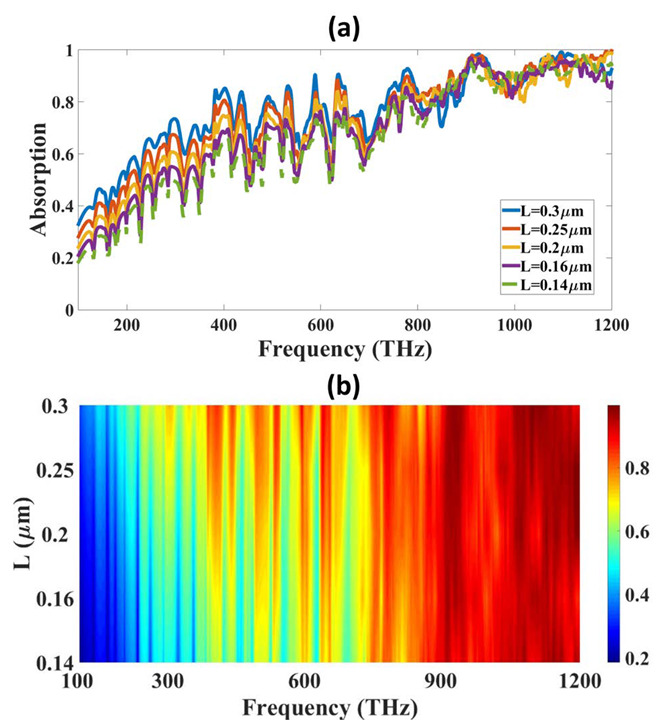
Variation in absorption for the different spectrum of L and frequency. (**a**) Line plot and (**b**) Contour plot. The red color at the top show the highest absorption of 1.

The optimization is carried out in the structure using parametric optimization and the behavior of the results is nonlinear, so nonlinear optimization is used and the results are presented in the figures. The values of the absorption L vary between 0.14 µm to 0.3 µm and variation results are presented in Figure 2. The absorption plot of Figure 2a shows that the absorption of L = 0.3 µm shows the highest absorption compared to all the other investigated ranges. The absorption of other L values shows less absorption. The lowest L value of 0.14 µm shows the lowest absorption for all the investigated ranges.

**Figure 3:**
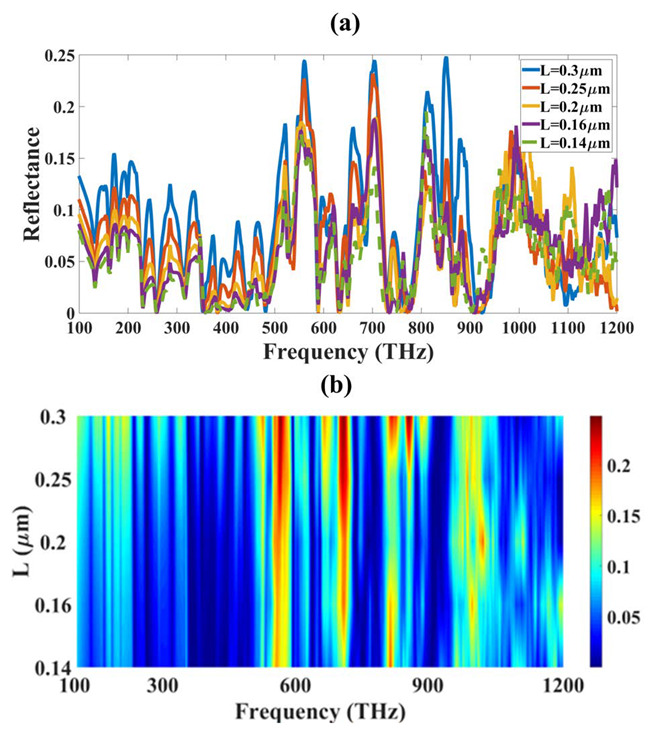
Variation in reflectance for the different spectrum of L and frequency. (**a**) Line plot and (**b**) Contour plot.

**Figure 4:**
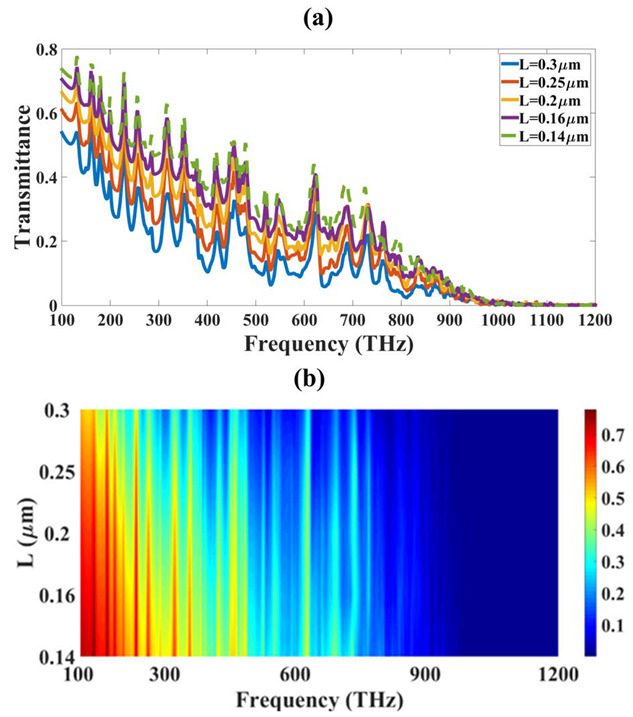
Variation in transmittance for the different spectra of L and frequency. (**a**) Line plot and (**b**) Contour plot.

Similarly, Figure 3 and Figure 4 show the variation in reflectance and transmittance for the different values of L. The absorption spectrum becomes wide over the entire frequency spectrum band for the larger values of the L. We have given line plots and contour plots in all the figures. The (a) part of the figure is showing a line plot and (b) part of the figure shows the contour part. The opposite response is also observed in transmittance, where the values are lower for the same range where absorption is higher. The absorption results of Figure 3 clearly show that the absorption of the higher THz region that is from 600 THz onwards is improving, and the high absorption is visible with red color which indicates a higher absorption of around 90% or higher. The absorption for the below 600 THz is showing small peaks of absorption and the absorption is increasing with an increase in the L size. The initial L value of 0.14 µm show that there is less absorption and increasing it to 0.3 µm increases it and there are more high absorption peaks in the higher values of L. The transmittance of the same variation is showing opposite results which indicates that the higher values of L decrease the transmittance which shows that all the light is trapped inside the absorber structure. The reflectance results show that there is very less reflectance for all the variations so there is not much effect of the variation in the reflectance results. The optimization is carried out with the nonlinear optimization method and the results for this optimization are presented in Figure 3 and Figure 4. The optimization for different parameters like L for absorption, reflectance, and transmittance show that the optimized value for good absorption is 300 nm.

**Figure 5:**
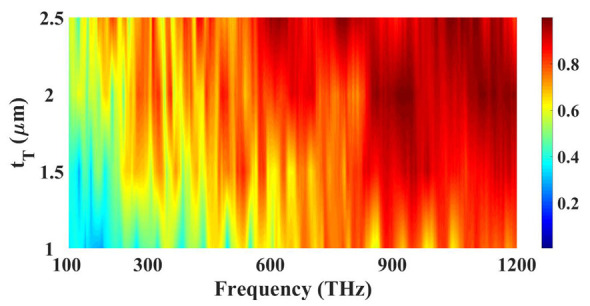
Absorption variation for the different height values of the tungsten layer. The red color at the top show the highest absorption 1.

Figure 5 shows the absorption variation for the variation in tungsten height values. The low amplitude of the absorption is observed for the small tungsten height, where the large band of light trapping is possible with the tick layer of the tungsten. The increase in height of the tungsten layer increases the overall ground layer structure which reduces the transmittance of the light through that structure which in a way increases the absorption of the structure which is visible from the result presented in Figure 5. The absorption is higher for the higher values of the tungsten thickness. There are more high peaks around the higher tungsten thickness of 2.5 µm. The lower tungsten thickness shows a lower response with the absorption being very low till 600 THz for these lower thickness values. The results of this variation are achieved because the higher height of the tungsten layer will enhance the ground plane height which will absorb all the light in it and not pass the light through that ground plane which is made up of tungsten material in this research. Different layer thicknesses of the structures are also optimized to show improvement in absorption. The absorption is optimized for the thickness of the tungsten ground layer, substrate layer, and gold resonator layer. From the plot, it is clear that the increase in thickness increases the absorption and higher absorption is achieved for 2500 nm thickness; thus, the optimized value is 2500 nm.

**Figure 6:**
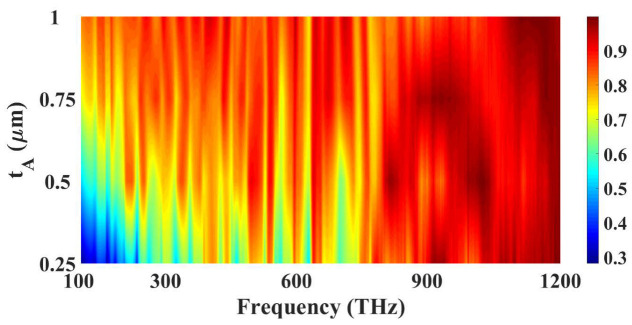
Absorption variation for the different height values of the gold resonator layer. The red color at the top show the highest absorption 1.

**Figure 7:**
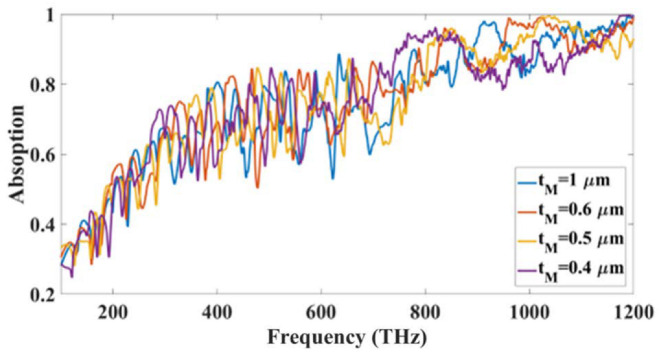
Absorption variation for the different height values of the MgF_2_ layer.

Similarly, Figure 6 show variation in the absorption for different gold layer thickness. The contour plot presented in the figure clearly shows that the variation is carried out for 250 nm to 1000 nm and the blue color results for 250 nm show that absorption is less for lower gold thickness and as the thickness is increased from 250 nm to 1000 nm than the absorption is increased, and the absorption is highest for 1000 nm thickness which is considered the optimized value for gold resonator layer. Figure 7 show the absorption line plot for different height of the MgF_2_ layer. The substrate thickness is important because the increase in substrate thickness absorbs more energy in it. We have investigated the substrate thickness from 400 nm to 1000 nm. We started from 400 nm because the substrate used in this design is a thick substrate whose values should be concerning the resonator and ground plane. The lesser values than 400 nm will make it a thin layer sandwiched between the resonator and substrate. The contour plot shows that an increase in substrate thickness is increasing the absorption level and the optimized value of it is considered 1000 nm because it is giving the highest absorption which is visible with the red color region near that thickness. As seen in Figure 5, Figure 6 and Figure 7, the absorber’s different components resonate at various frequencies. The design dimensions, in terms of height and breadth, significantly impact the values of a structure’s absorption. Resonance is produced on the surface in several different places due to the dipole moment. Only the gold resonator will resonate at particular frequencies; the tungsten and MgF_2_ surfaces will enable light absorption while minimizing the influence of reflection. Because of this, the force of the electric field trapped by the various structural edges is significantly increased. Stronger dipole moments at edges contribute to increased absorption that results from increased light-trapping effects.

**Figure 8:**
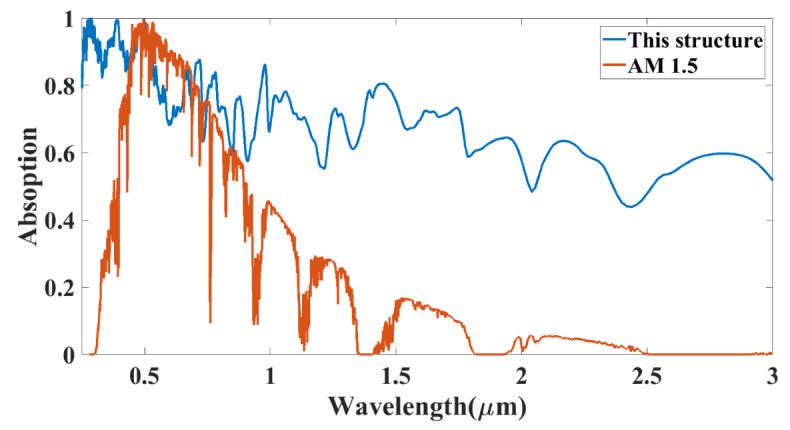
Comparative response of the absorption with proposed structure and normalized AM 1.5 values.

Figure 8 shows absorption for the proposed structure with the absorption spectrum offered by AM 1.5 [30,31,32] spectral irradiance in the wavelength domain. The results are converted in the wavelength to show the comparison of the results with the AM 1.5 irradiance curve. The results show that most of the energy of the visible and UV spectra is absorbed in the proposed structure. It can achieve a high absorption efficiency for visible and UV spectra while somewhat low for infrared spectra. Figure 8 will give the relative comparative analysis for comparing the actual infrared and solar radiation absorption spectrum. The absorption values achieve complete absorption in the range of wavelengths from 0.25 to 1 µm (300 to 1200 THz). The remaining portion of the bigger wavelength’s range, which spans from 1 to 3 µm (100–300 THz), will see the absorption reach its lowest point of 20% and its highest point of 80%. In this layered structure of the design for the solar absorber, absorption is trapped in the hybrid structure. It is observed in the proposed solar absorber structure that the wideband absorption behavior of this device will also absorb the near and far infrared wavelength. For the higher infrared region as the AM 1.5 spectral energy is less so the decrease in absorption results does not affect much in the overall absorption of the solar energy.

**Figure 9:**
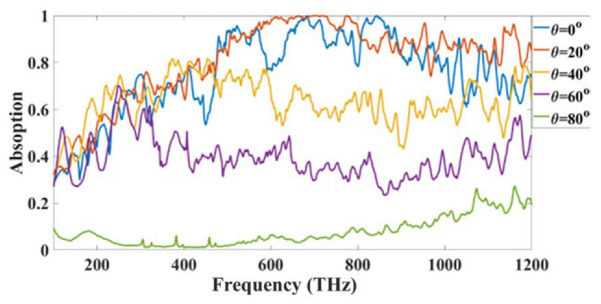
Absorption variation for the different oblique angles of the incident.

Figure 9 shows the change in the results for the large oblique angle of incident values. The results are analyzed for different angles and the design stability is achieved for a wide angle of incidence. Only for the higher angle values of 80 degrees, the results are low otherwise for all the other angles the results are more than 50% for the entire range. The angle of incidence is crucial while designing the solar absorber structure as the sun moves around the day and there is sun radiation is different at different angles the absorption presented in Figure 9 clearly shows that the absorption is high for all the angles except one angle where it is around 20% so absorber shows the wide angle of incidence which improve the solar absorber efficiency overall.

In the end, the novelties of our proposed design compared to other previously published designs.

- The structure is gold–MgF2–tungsten with MgF_2_ and tungsten layers which can be fabricated easily while the gold material is having few square slots which create the shape which is shown. Which can easily be achieved through lithography. Firstly, the design fabrication is easy and possible using CVD and the lithography technique.
- The materials used, like MgF_2_ and tungsten, are easily available and their cost is also low.
- The absorber is showing good performance in the UV and visible regions and absorbs most of the energy of these two regions as shown in the AM 1.5 comparison plot.
- The design is optimized for different geometrical parameters.
- The absorption is also compared with similarly published designs in Table 2 which show that our design performs well compared to other designs.

The proposed structure is also presented with other such structures and their results in Table 2. The comparative analysis includes unit cell dimension, metal layers, maximum absorption, bandwidth, and operating frequency. It is found that the design has a good absorption response with a wide angle of incidence, which can be adjustable over the frequency band by adjusting the height of the different layers. This absorption spectrum is susceptible to additional modification due to many factors, such as the height of the layers and the angle at which the incident values are measured.

The proposed gold resonator based on an MgF_2_ substrate with a back tungsten layer can be fabricated using a chemical vapor deposition (CVD) [33]. The plasmonic structure can also be fabricated using a lithography [34]. E-beam lithography and CVD may also be used to create the structure. The mask of the resonator can be used to create the resonator shape using the CVD technique. Thus, the structure can be fabricated using both techniques.

## 4. Conclusions

The cross and squared composited gold resonator and the MgF_2_-tungsten-layer-based wideband solar absorber are both subjected to computational analysis to study their performance in the presence of a solar spectrum with a spectrum of 0.5 to 3 µm. Using the finite element technique (FEM), a numerical investigation of the solar cell is carried out. Values of transmittance, reflectance, and absorption are explored to gain insight into the absorber’s behavior. Furthermore, nonlinear parametric optimization is researched for the different parameters to determine the high absorption values for the analyzed spectra. To determine the extent of the absorption discrepancy between the calculated results and the normalized AM 1.5 spectrum, the calculation results are compared. At the shorter wavelength, the suggested structure has an efficiency of 98%, but at the longer wavelength, it offers average efficiency of 73%. In addition, the suggested structure has been observed to have good absorption stability up to an oblique input incidence angle of 60° with an average of 50% absorption. The suggested results can be used to assist in constructing high-efficiency solar cells, which will benefit various solar applications.

## Figures and Tables

**Table 1:** Optimized Parameters for design construction.

Parameter	Size (nm)
t_A_	1000
L	300
t_M_	1000
t_T_	2500
W	2000
P	1000

**Table 2:** The proposed structure results in a comparison of bandwidth, frequency, absorption, material, and size.

Ref.	Unit Cell Size (um^2^)	No. of Layers	Approximate Maximum Absorption	Bandwidth (THz)	Operating Frequency (F_L_) (THz)	Operating Frequency (F_H_) (THz)
This structure	2 × 2	Tungsten-MgF_2_-Gold	~95%	380	723	1103
[35]	0.48 × 0.48	Metal-tungsten-Si-Ag	~95%	155.74	272.53	428.27
[36]	0.9 × 0.9	Graphene-PMMA-Ag-SiO_2_	~71.1%	449.69	299.79	749.48
[37]	0.32 × 0.32	Dielectric-meta	~90%	37.789	176.348	214.137
[38]	0.3 × 0.5	Au	~95%	41.64	124.91	166.55
[39]	0.34 × 0.34	SiO_2_-T-silica	~80	896.33	119.91	1016.24
[40]	0.3 × 0.3	Dielectric-meta-Au	~99%	499.65	99.93	599.58
[41]	70 × 70	AI-dielectric	~98%	0.8	0.8	1.6
[42]	0.4 × 0.4	Ag-graphene-SiO_2_-Ag	~80	291.46	374.74	666.20
[43]	0.53 × 0.53	Gold-glass-GaAs-gold	~70	97.82	537.91	635.73
[44]	0.6 × 0.6	Dielectric-Au-MgF_2_	~99%	80	140	220
[45]	-	AI-dielectric-Au	~99%	180	100	280
[46]	0.3 × 0.3	Dielectric-metal-Au	~98%	40	90	130
[47]	2.5 × 2.5	Silica-graphene-dielectric	~20%	4.996	2.498	7.494
[30]	1 × 1	Gold-SiO_2_-Gold	~94%	200	550	750

## Data Availability

The data will be made available at a reasonable request to the corresponding author.
